# The efficacy and safety of neoadjuvant immunotherapy in resectable locally advanced esophageal squamous cell carcinoma: A systematic review and meta-analysis

**DOI:** 10.3389/fimmu.2023.1118902

**Published:** 2023-02-17

**Authors:** Wenwu He, Chenghao Wang, Changding Li, Xin Nie, Haojun Li, Jialong Li, Na Zhao, Haijun Chen, Xiaojie Miao, Yongtao Han, Lin Peng, Xuefeng Leng

**Affiliations:** ^1^ Department of Thoracic Surgery, Sichuan Cancer Hospital and Institute, Sichuan Cancer Center, Cancer Hospital Affiliated to University of Electronic Science and Technology of China, Chengdu, China; ^2^ Medical Affairs Department of BeiGene (Beijing) Co., Ltd, Beijing, China

**Keywords:** neoadjuvant immunotherapy, esophageal squamous cell carcinoma, nICRT, nICT, meta-analysis

## Abstract

**Objective:**

This systematic review and meta-analysis aimed to explore the efficacy and safety of neoadjuvant immunotherapy in patients with resectable locally advanced esophageal squamous cell carcinoma (ESCC).

**Background:**

Several studies have reported the outcomes of neoadjuvant immunotherapy in patients with ESCC. However, phase 3 randomized controlled trials (RCTs) with long-term outcomes and the comparison of different therapeutic strategies are lacking.

**Methods:**

Studies involving patients with advanced ESCC treated with preoperative neoadjuvant immune checkpoint inhibitors (ICIs) were searched through PubMed, Embase, and Cochrane Library up to July 1, 2022. The outcomes were presented as proportions and pooled respectively by fixed or random effect model depending on the heterogeneity between studies. All analyses were performed using the R packages meta 5.5-0 and meta-for 3.4-0.

**Results:**

Thirty trials involving 1406 patients were included in the meta-analysis. The pooled pathological complete response (pCR) rate for neoadjuvant immunotherapy was 0.30 (95% confidence interval [CI]: 0.26–0.33). The pCR rate of neoadjuvant immunotherapy combined with chemoradiotherapy (nICRT) was significantly higher than that of neoadjuvant immunotherapy combined with chemotherapy (nICT) (nICRT: 0.48, 95% CI: 0.31–0.65; nICT: 0.29, 95% CI: 0.26–0.33; *p*=0.03). No significant difference in efficacy was observed between the different chemotherapy agents and treatment cycles. The incidences of grade 1–2 and 3–4 treatment-related adverse events (TRAEs) were 0.71 (95% CI: 0.56–0.84) and 0.16 (95% CI: 0.09–0.25), respectively. Patients treated with nICRT and carboplatin had a higher incidence of grade 3–4 TRAEs compared with those treated with nICT (nICRT: 0.46, 95% CI: 0.17–0.77; nICT: 0.14, 95% CI: 0.07–0.22; *p*=0.03) and cisplatin (carboplatin: 0.33, 95% CI: 0.15–0.53; cisplatin: 0.04, 95% CI: 0.01–0.09; *p*<0.01).

**Conclusion:**

Neoadjuvant immunotherapy has good efficacy and safety profiles in patients with locally advanced ESCC. Additional RCTs with long-term survival data are warranted.

## 1 Introduction

According to the 2020 global tumor data, esophageal cancer (EC) is the seventh most common malignant tumor worldwide, and its mortality rate ranks sixth (1). Esophageal squamous cell carcinoma (ESCC) and adenocarcinoma are the two main histological subtypes of EC. Adenocarcinoma is the main subtype in the United States and Europe, accounting for approximately 70% of cases, whereas ESCC is the main subtype in Asia, accounting for approximately 90% of the incidence of EC. More than half of EC patients are already at locally advanced stage when diagnosed. Neoadjuvant therapy such as neoadjuvant chemoradiotherapy or chemotherapy has been the standard treatment for locally advanced EC. However, the postoperative recurrence and metastasis rates of locally advanced EC are still high after neoadjuvant therapy, ranging from 30% to 50%. Besides, it has been a bottleneck to further improve the treatment outcome in this patient population (2, 3). In terms of pathology, esophageal adenocarcinoma and ESCC are two completely different pathological types (4). This study focused on the perioperative treatment of locally advanced ESCC. The perioperative treatment of locally advanced ESCC has become a research hotspot in recent years. Based on the results of the CROSS (5, 6), NEOCRTEC5010 (7, 8), and JCOG1109 (9) trials, neoadjuvant chemoradiotherapy (nCRT) or neoadjuvant chemotherapy (nCT) combined with surgery are the standard treatment options for locally advanced ESCC. However, the 5-year overall recurrence rate of ESCC remains high (8), and its long-term survival rate is poor (6). To date, there is no clear evidence to support a significant difference in survival benefits between patients receiving nCRT and nCT (10, 11).

In recent years, immunotherapy, represented by immune checkpoint inhibitors (ICIs), has excellent efficacy and controllable toxicity in advanced EC, according to the results from ESCORT, ATTRACTION-3, KEYNOTE-181, KEYNOTE-590 and CheckMate648 studies (12–16). An increasing number of researchers have begun to combine ICIs with nCRT or nCT for the treatment of locally advanced ESCC. With the completion of phase 2 clinical studies, early results have reported the efficacy and safety of neoadjuvant immunotherapy combined with chemoradiotherapy (nICRT) and neoadjuvant immunotherapy combined with chemotherapy (nICT). However, the superiority of this combined strategy remains uncertain owing to insufficient sample sizes in individual phase 2 trials, varying clinical study designs, and the lack of randomized controlled phase 3 trials (RCTs) with long-term outcomes. Moreover, the effects of different chemotherapy agents and cycle numbers remain uncertain.

Therefore, we conducted a systematic review and meta-analysis to evaluate the antitumor efficacy and safety of nICRT and nICT. We aimed to obtain more accurate conclusions and summarize nICRT and nICT as reliable evidence for preoperative neoadjuvant therapy for locally advanced ESCC to facilitate clinical decision-making and the development of future randomized controlled phase 3 clinical trials.

## 2 Methods

This systematic review and meta-analysis followed the Preferred Reporting Items for Systematic Reviews and Meta-Analyses (PRISMA) statement (17). The study was registered with the International Prospective Register of Systematic Reviews database (ID: CRD42022355086), and the protocol can be found there.

### 2.1 Search strategy and selection criteria

PubMed, Embase, and Cochrane Library were searched for relevant publications up to July 1, 2022. All keywords were searched using Medical Subject Headings. The detailed search strategy is presented in **Supplementary Table 1**. Databases were searched for titles and abstracts.

The inclusion criteria were as follows (1): prospective or retrospective studies involving patients with ESCC treated with preoperative neoadjuvant ICIs and (2) studies reporting at least one of the following primary outcomes: pathological complete response (pCR, defined as no residual tumor cells), major pathological response (MPR, defined as < 10% residual tumor cells), objective response rate (ORR), disease control rate (DCR), downstaging rate, R0 resection rate, incidence of treatment-related adverse events (TRAEs), and incidence of anastomotic leakage.

The exclusion criteria were as follows (1): case report, review, commentary, or conference abstract and (2) for multiple articles published with overlapping or repeating data, those reporting the most comprehensive data.

### 2.2 Data extraction

Two reviewers (CHW and CDL) independently reviewed and extracted the necessary data from the selected articles. Disagreements were resolved through discussion or by a third researcher (WWH) to decide whether to include the study. An Excel spreadsheet was used to record the data.

The following terms were extracted or summarized as proportions from the included articles if available: first author name, journal, year of publication, author countries, study type, study phase, study center, main inclusion criteria, study arms, sample size, efficacy evaluation criteria, sex, age, Eastern Cooperative Oncology Group Performance Status (ECOG PS), body mass index, tumor location, primary tumor length, programmed cell death ligand-1 (PD-L1) expression levels, PD-L1 cutoff value, clinical tumor-node-metastasis (TNM) tumor stage, tumor stage criteria, surgical method, number of dissected lymph nodes, extent of lymph node dissection, R0 resection rate, ICI drugs, ICI dose, chemotherapy drugs, chemotherapy dose, number of surgical resection, MPR, pCR, complete response (CR) rate, partial response rate, stable disease rate, progressive disease rate, downstaging rate, ypTNM, efficacy analysis sample size, follow-up time, 1/3-year overall survival (OS) rate, 0.5-/1-/3-year disease-free survival (DFS) rate, incidence of grade 1–2 TRAEs, incidence of grade 3–4 TRAEs, incidence of immune-related adverse events, surgical delay rate, incidence of anastomotic leakage, in-hospital mortality rate, and 30-day mortality rate.

### 2.3 Quality assessment

The majority of included studies were single-arm studies, and the Methodological Index for Nonrandomized Studies (MINORS) checklist was used to assess the quality of these studies (18). Items were scored as 0 (not reported), 1 (reported but inadequate), or 2 (reported adequately). The ideal scores were 16 and 24 for non-comparative and comparative studies, respectively.

### 2.4 Statistical analyses

The outcomes (pCR, MPR, R0 resection rate, CR rate, ORR, DCR, downstaging rate, anastomotic leakage, grade 1–2 TRAE rate, and grade 3–4 TRAE rate) are presented as proportions. All analyses were performed using the R package *meta* 5.5-0 and *meta-for* 3.4-0. The proportions were first obtained through a double arcsine transformation, which works pretty well for normalizing and variance-stablizing the sampling distribution of proportions, and then pooled using the inverse variance method in meta analysis. Heterogeneity was evaluated using the *Q*-test and statistical inconsistency index (*I^2^*) for each outcome. When *p* was <0.05 (for the *Q*-test) or *I^2^* was >50%, the studies included were considered heterogeneous and the random-effect model was applied. Otherwise, a fixed-effects model was used for analysis. Subgroup analyses were performed based on study type (prospective vs. retrospective), combination treatment (nICT vs. nICRT), neoadjuvant treatment cycles (2 cycles vs. ≥2 cycles), platinum type (carboplatin vs. cisplatin), and taxane type (paclitaxel vs. nab-paclitaxel) if there were at least two studies included in each subgroup. The common chemotherapy frequency of nICRT is weekly, which is different from that of nCRT; therefore, subgroup analysis by neoadjuvant treatment cycles did not include studies of nICRT. The outcomes in each subgroup were synthesized and the results were compared across subgroups by statistical test (19). Corresponding *p* values for the comparison were reported along with the results of subgroup analyses. A common estimate of between-study heterogeneity was assumed when the number of studies in a subgroup was ≤5. Sensitivity analyses were conducted using the leave-one-out method. The pooled effect and corresponding *I^2^* were recalculated each time, leaving out one study. Thus, studies that affect the results can be identified, and the robustness of the results can be further evaluated. Funnel plots were created, and Egger regression tests were performed to evaluate publication bias if the results were synthesized from more than 10 studies. *P <*0.05 was considered statistically significant for all analyses.

## 3 Results

### 3.1 Trial characteristics

**Figure 1:**
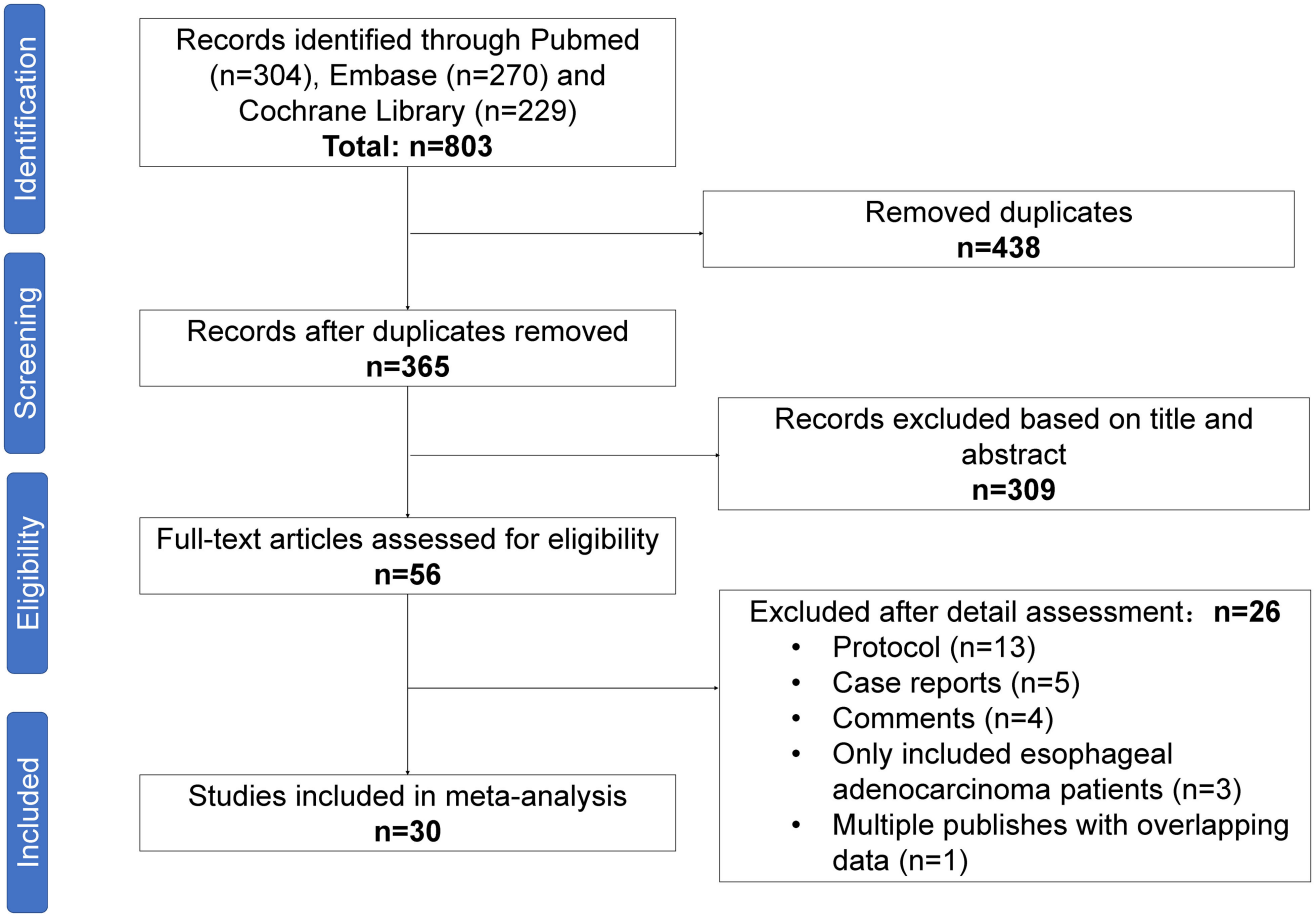
Preferred Reporting Items for Systematic Reviews and Meta-Analyses (PRISMA) diagram of the study selection.

A PRISMA diagram of the study selection procedure is shown in **Figure 1**. In total, 803 articles were identified based on the search strategy, and 30 trials with 1406 patients were eligible for inclusion in the final meta-analysis. Most studies were conducted in China. Among the 30 included studies, 13 were single-arm prospective, one was a dual-arm prospective, and 16 were retrospective. Twenty-seven studies used nICT as neoadjuvant therapy, two studies examined nICRT, and one study included both nICT and nICRT. All studies used programmed cell death protein 1 (PD-1) inhibitors, and only one retrospective study included patients who received PD-L1 inhibitors. The most common nCT regimens were paclitaxel or nab-paclitaxel plus carboplatin or cisplatin. The main characteristics of the included studies are presented in **Table 1**, and the primary outcomes are shown in **Table 2**.

**Table 1:** Characteristics of included studies.

First author (Year) Country	Study type	Study center	Sample size	Clincial stage	Age (Median)	Male (%)	ECOG (0/1/2)	Tumor location (U/M/L/EGJ)	cT (1/2/3/4)	cN (0/1/2/3)	Turmor stage (II/III/Iv)	Intervention	ICI	CT regimen	cycles of nICT
Dijian Shen(2021)/China	One-arm prospective	Single	28	cT1N1-3M0 or cT2-4aN0-3M0	62.2	0.96	0.89/0.11/0	0.11/0.50/0.29/0.11	0/0.11/0.82/0.07	0.07/0.54/0.32/0.07	0.14/0.75/0.11	nICT	Nivo/Pemb/Camr	CP+ABX	2
Weixiong Yang (2022)/China	One-arm prospective	Single	23	II-III	58.6	0.96	0.91/0.09/0	0.04/0.39/0.57/0	NR	NR	0.35/0.63/0	nICT	Camr	CP+ABX	2
Wenqun Xing (2021)/China	two-arm prospective	Single	15	II/III/IVa	63.8	0.87	0.53/0.47/0	NR	0/0/0.60/0.40	0.27/0.53/0.20/0	0.20/0.47/0.33	nICT	Tori	DDP+PTX	2
			15	II/III/IVa	63.13	0.60	0.33/0.67/0	NR	0/0/0.87/0.13	0.20/0.53/0.27/0	0.20/0.67/0.13	nICT	Tori	DDP+PTX	2
Zhenyang Zhang (2021)/China	One-arm prospective	Single	30	cT3-T4aN0-3M0 or cT1-2N1-3M0	58.3	0.87	0.17/0.83/0	0.07/0.60/0.33/0	0/0/0.90/0.10	0.00/0.40/0.60/0	0/0.9/0.1	nICT	Sint	DDP+ABX	2
Peng Yang (2021)/China	One-arm prospective	Single	16	cT1N1-3M0 or cT2-4aN0-3M0	60.5	0.88	0.81/0.19/0	0.19/0.50/0.31/0	0/0.13/0.81/0.06	0.13/0.63/0.19/0.06	0.25/0.63/0.13	nICT	Camr	CP+PTX	2
Hongtao Duan (2021)/China	One-arm prospective	Multicenter	23	T2-xNxM0	63.5	0.91	0.91/0.09/0	0.04/0.83/0.13/0	0/0.04/0.87/0.09	0.22/0.52/0.26/0	0.17/0.74/0.09	nICT	Sint	NDP+ABX+D	3
Hongtao Duan (2022)/China	One-arm prospective	Single	18	T2-4aNxM0	64	0.78	NR	0.00/0.83/0.17/0	0/0/0.94/0.06	0.39/0.50/0.11/0	0.39/0.56/0.06	nICT	Pemb	NDP+ABX/D	3
Xiaolong Yan (2022)/China	One-arm prospective	Single	45	T2-4aNxM0	63.8	0.60	NR	NR	0/0.16/0.76/0.09	0.44/0.33/0.18/0.04	0.40/0.53/0.07	nICT	Tisl	CP+ABX	3
Wenwu He (2022)/China	One-arm prospective	Single	20	T3-4aN1-3M0	62.1	0.75	NR	0.00/0.70/0.30/0	NR	NR	0/0.80/0.20	nICT	Tori	CP+PTX	2
Lei Gao (2022)/China	One-arm prospective	Single	20	≥ cT3 or ≥ N+	58.3	0.85	NR	0.10/0.65/0.25/0	NR	NR	NR	nICT	Tori	DDP+D	2
Jun Liu (2022)/China	One-arm prospective	Multicenter	56	T2N1-3M0/T3N0-3M0/T4N0-3M0	61	0.75	0.70/0.30/0	0.02/0.48/0.50/0	0.02/0.25/0.68/0.04	0.16/0.39/0.38/0.07	0.23/0.68/0.09	nICT	Camr	DDP+ABX	2
Jun Liu (2022)/China	One-arm prospective	Multicenter	60	T1b-4a, N2-3 (≥3 stations), and M0-1	65	0.83	0.95/0.05/0	0.15/0.60/0.25/0	0/0.15/0.78/0.07	0.00/0.00/0.92/0.08	0/0.85/0.15	nICT	Camr	DDP+ABX	2
Liwei Xu (2022)/China	One-arm prospective	Single	37	T2–4aNanyM0 or T1N1–3M0	63.3	0.95	NR	NR	NR	NR	0.27/0.73(III-IV)	nICT	Camr	CP+ABX	NR
Chengqiang Li (2021)/China	One-arm prospective	Single	20	T2-4a Nany M0	62	0.95	0.15/0.85/0	0.25/0.55/0.20/0	0/0/0.80/0.20	0.10/0.25/0.50/0.15	0.1/0.65/0.25	nICRT	Pemb	CP+PTX	2
Guozhen Yang (2021)/China	Retrospective	single	12	T2–3, N0–3	56	0.58	NR	0.08/0.50/0.42/0	NR	NR	0/0.17/0.67/0.17	nICT	Camr	ABX+S1	3
Xiao Ma (2022)/China	Retrospective	single	34	T3-4a N1-3 M0,	61	0.91	0.74/0.27/0	0/0.74/0.27/0	0/0/0.77/0.24	0/0/0.47/0.53	0/0/0.41/0.59	nICT	Pemb/Camr	DDP+PTX	2
Zhigang Wu (2021)/China	Retrospective	single	38	T3–4a, N1–3, M0	61	0.95	0/0.90/0.11	0.08/0.55/0.37/0	0/0.11/0.95/0.05	0/0.68/0.26/0.05	0/0/0.68/0.32	nICT	Camr/Pemb/ Sint	DDP or CP + D or PTX	≥1
Xinke Zhang (2022)/China	Retrospective	single	64	NR	NR	0.78	0.31/0.69/0	0.05/0.47/0.48/0	0/0.13/0.88(T3-4)	0.31/0.69(N1-2)/0	0/0.30/0.52/0.19	nICT	Camr	TP or FP	2or3
Bingjiang Huang (2021)/China	Retrospective	single	23	II–Iva	59.2	0.91	NR	0.17/0.57/0.26/0	0/0.17/0.65/0.17	0/0.17/0.61/0.22	0/0.13/0.61/0.26	nICT	Pemb	NDP+D	2
Huilai Lv (2022)/China	Retrospective	single	96	II–IVA	65	0.70	0.41/0.47/0.13	0.15/0.48/0.38/0	0/0.05/0.90/0.05	0.39/0.56/0.05/0	0/0.42/0.54/0.04	nICT	Sint	TP	2-4
Xin Xiao (2022)/China	Retrospective	single	57	T1-2N+M0 or T3-T4aN(any)M0,	66	0.83	NR	0.21/0.53/0.26/0	0/0.05/0.75/0.19	0.14/0.65/0.19/0.02	0/0.18/0.63/0.19	nICT	PD-1	DDP+PTX	2
Yongkui Yu (2022)/China	Retrospective	single	79	T2N+M0-T3-4N0/+M0	62.05	0.73	NR	0.16/0.67/0.16/0	0/0.15/0.70/0.15	0.34/0.66(N1-3)	0/0.04/0.94/0.03	nICT	PD-1	DDP or NDP + PTX	2
Jiahan Cheng (2022)/China	Retrospective	single	40	T1N+M0 or T2-4aN0-3M0	64.3	0.75	0.78/0.23/0	0.30/0.43/0.28/0	0/0.05/0.93/0.03	0.03/0.33/0.63/0.03	0/0.05/0.93/0.03	nICT	PD-1	DDP+PTX or 5-FU	2-4
Zhi-Nuan Hong (2021)/China	Retrospective	single	38	cT1-2N1-3M0 or cT3-4aN0-3M0	58.8	0.58	NR	0.03/0.55/0.42/0	NR	NR	NR	nICT	Camr/Pemb/ Sint	DDP+ABX	2-4
Zhi‑Nuan Hong (2022)/China	Retrospective	single	27	cT1N1-3M0 or cT2-4aN0-3M0;	58.9	0.82	NR	0.04/0.63/0.33/0	NR	NR	0/0.19/0.44/0.37	nICT	Camr/Pemb/ Sint	TP	2-4
Ye-Han Zhou (2022)/China	Retrospective	single	14	NR	NR	0.64	NR	0.07/0.86/0.07/0	0/0/1.00/0	0.07/0.57/0.29/0.07	NR	nICT	Tori	CP+PTX	2
Yang Yang (2022)/China	Retrospective	multicenter	41	NR	61	0.83	0.63/0.29/0.07	0.07/0.73/0.20/0	0/0.1/0.90/0	0.10/0.73/0.17/0	0/0.20/0.81/0.00	nICI	PD-1 and SHR-1316	none	NR
			299	NR	64	0.83	0.78/0.20/0.02	0.14/0.43/0.40/0.03	0.02/0.10/0.77/0.11	0.10/0.39/0.48/0.04	0.02/0.13/0.69/0.16	nICT		TP or FP	NR
			30	NR	62	0.93	0.70/0.30/0	0.23/0.53/0.17/0.07	0/0.03/0.70/0.27	0.10/0.27/0.43/0.20	0.03/0.1/0.53/0.33	nICRT		TP or FP	NR
Seong Yong Park (2020)/Korea	Retrospective	single	16	T1N1-2 or T2-4aN0-2	58.5	0.81	NR	0.25/0.38/0.38/0	0.25/0.19/0.50/0.06	0.13/0.31/0.38/0.19	NR	nICRT	Pemb	CP+PTX	2
Guo-Qiang Yin (2022)/China	Retrospective	single	34	cT1N1-3M0 or cT2-4aN0-3M0	59	0.88	0.82/0.18/0	0.12/0.53/0.35/0	0/0.12/0.79/0.09	0.09/0.53/0.35/0.03	0/0.15/0.77/0.09	nICT	Camr	CP+PTX	2-4
Yi-Min Gu (2022)/China	Retrospective	single	38	T1N+M0 or T2–4aN0–3M0	66	0.71	0.76/0.18/0.05	NR	0/0.08/0.92/0	0.24/0.13/0.63/0	0/0.24/0.76/0.00	nICT	PD-1	DDP+PTX	2-4

nICT, immune checkpoint inhibitor in combination with chemotherapy; nICRT, immune checkpoint inhibitor in combination with chemotherapy and radiotherapy; Nivo, Nivolumab; Pemb, Pembrolizumab; Camr, Camrelizumab;

Tori, Toripalimab; Sint, Sintilimab; Tisl, tislelizumab; CP, carboplatin; DDP, Cisplatin; NDP, nedaplatin; ABX, albumin paclitaxel; PTX, paclitaxel; D, Docetaxel; TP, Platinum+Taxanes; FP, Platinum+Fluorouracil; NR, not report.

**Table 2:** Primary outcomes of included studies.

First author/Year	pCR	MPR	Down-staging rate	CR	ORR	DCR	R0 resection	Anastomotic leakage	Grade 1-2 TRAEs	Grade 3-4 TRAEs
Shen 2021	0.33	NR	NR	0.44	0.89	1.00	0.96	0.19	0.57	0.07
Yang 2022	0.25	0.50	0.65	0.05	0.91	1.00	1.00	0.10	NR	NR
Xing 2021	0.36	NR	NR	NR	NR	NR	1.00	0.09	NR	NR
	0.08	NR	NR	NR	NR	NR	1.00	0.08	NR	NR
Zhang 2021	0.17	0.52	NR	0.00	0.67	0.97	1.00	0.13	0.90	0.03
Yang 2021	0.31	NR	NR	0.25	0.81	1.00	0.94	NR	NR	NR
Duan 2021	0.35	0.53	0.77	0.38	0.63	0.88	0.94	0.12	NR	0.30
Duan 2022	0.46	0.69	0.69	0.22	0.50	0.94	0.85	0.08	NR	0.28
Yan 2022	0.50	0.72	0.75	NR	NR	NR	0.81	0.06	1.00	0.42
He 2022	0.19	0.44	0.72	0.33	0.61	1.00	0.88	0.00	0.80	0.20
Gao 2022	0.17	0.42	NR	0.00	0.70	0.95	1.00	0.08	NR	0.15
Liu 2022	0.31	0.59	0.77	0.14	0.67	1.00	1.00	0.06	0.75	0.11
Liu 2022	0.39	0.69	NR	0.06	0.49	0.96	0.98	0.10	0.97	0.57
Xu 2022	0.22	0.49	NR	NR	NR	NR	NR	NR	0.85	0.15
Li 2021	0.56	0.89	NR	0.53	0.95	0.95	0.94	0.06	NR	0.65
Yang 2021	0.33	0.42	0.83	0.00	0.58	1.00	1.00	0.17	1.00	0.00
Ma 2022	0.24	NR	0.68	NR	NR	NR	0.94	0.09	0.59	0.00
Wu 2021	0.34	0.42	NR	0.00	0.68	1.00	0.92	NR	NR	NR
Zhang 2022	0.42	NR	NR	NR	NR	NR	NR	NR	NR	NR
Huang 2021	0.33	NR	NR	0.30	0.87	0.96	1.00	NR	NR	NR
Lv 2022	0.30	0.63	0.74	NR	NR	NR	0.99	0.05	0.51	0.13
Xiao 2022	0.32	NR	NR	NR	NR	NR	0.97	0.05	NR	NR
Yu 2022	NR	NR	NR	NR	NR	NR	NR	NR	NR	NR
Cheng 2022	0.38	NR	NR	NR	NR	NR	NR	0.03	0.90	0.03
Hong 2021	0.13	0.42	NR	NR	NR	NR	1.00	0.08	NR	NR
Hong 2022	0.19	NR	NR	NR	NR	NR		0.11	0.63	NR
Zhou 2022	NR	NR	NR	NR	NR	NR	NR	NR	NR	NR
Yang 2022	0.12	NR	NR	NR	NR	NR	0.98	0.00	0.22	0.07
	0.26	NR	NR	NR	NR	NR	0.98	0.06	0.39	0.14
	0.42	NR	NR	NR	NR	NR	0.98	0.04	0.63	0.30
Park 2020	NR	NR	NR	NR	NR	NR	1.00	0.19	NR	NR
Yin 2022	0.35	0.50	0.79	0.06	0.62	1.00	0.94	NR	NR	NR
Gu 2022	0.24	NR	0.73	NR	NR	NR	0.97	0.05	0.16	0.03

pCR, pathological complete response; MPR, major pathological response; CR, complete response; ORR, overall response rate; DCR, disease control rate; TRAEs, treatment-related adverse events; NR, not reported.

### 3.2 Quality assessment

The quality of all included studies was judged according to the MINORS. Of the 30 included studies, six were designed as comparative studies. For these comparative studies, the total score was 16–22. The scores of non-comparative studies ranged from 9 to 15. As the aim of the included studies was to evaluate the efficacy and safety of neoadjuvant ICI therapy, the primary endpoints of most studies were safety or short-term efficacy endpoints, such as pCR and MPR. Follow-up data were not reported in 17 of the 30 studies. Generally, the included studies were of high quality and suitable for meta-analysis. The details of the quality assessment of all the studies are presented in **Supplementary Table 2**.

### 3.3 Efficacy outcomes

#### 3.3.1 Pathological response

**Figure 2:**
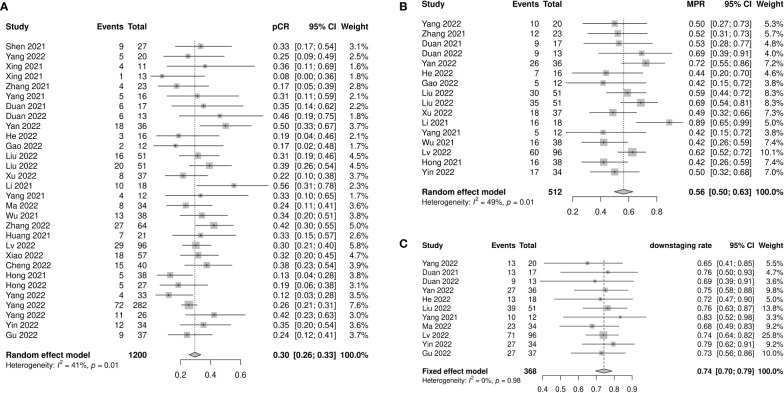
Pathological response of neoadjuvant immune checkpoint inhibitors therapy. **(A)** Pathological complete response, **(B)** major pathological response, and **(C)** downstaging rate.

pCR was reported in 26 of the 1200 patients. The pooled pCR was 0.30 (95% confidence interval [CI]: 0.26–0.33, *I^2^=*41%, *p*=0.01) (**Figure 2A**). Subgroup analyses results showed that the pCR rate of the nICRT group was significantly higher than that of the nICT group (nICRT: 0.48, 95% CI: 0.31–0.65; nICT: 0.29, 95% CI: 0.26–0.33; *p*=0.03). No significant differences in pCR were observed in the subgroup analyses according to study type, neoadjuvant treatment cycles, platinum type, or taxane type (**Supplementary Figure 1**).

Sixteen studies with 512 patients reported MPR rates ranging from 0.42 to 0.89. The pooled MPR was 0.56 (95% CI: 0.50–0.63, *I^2^=*49%, *p*=0.01) (**Figure 2B**). The downstaging rate was 0.74 (95% CI: 0.70–0.79, *I^2^=*0%, *p*=0.98) (**Figure 2C**), pooled from 11 studies with 368 patients. Subgroup analyses showed no significant differences in MPR or downstaging rate among study type, neoadjuvant therapy cycle number, platinum type, or taxane type (**Supplementary Figures 2**, **3**).

#### 3.3.2 Radiological response

**Figure 3:**
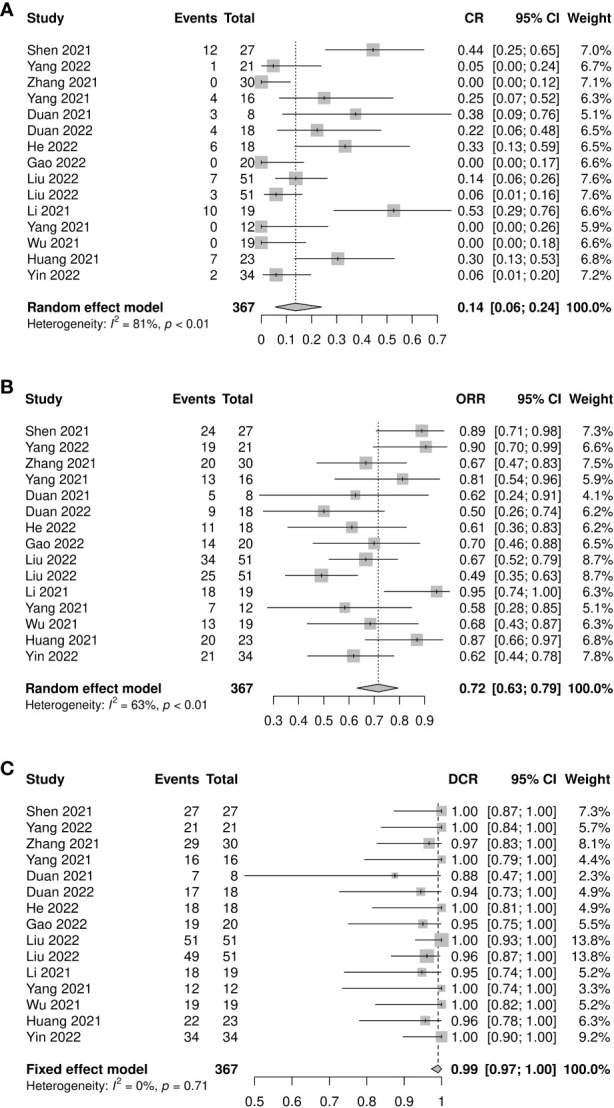
Radiological response of neoadjuvant immune checkpoint inhibitors therapy. **(A)** Complete response rate, **(B)** objective response rate, and **(C)** disease control rate.

A total of 367 patients from 15 studies were included in the analysis of CR rate, ORR, and DCR. The pooled CR rate, ORR, and DCR were 0.14 (95% CI: 0.06–0.24, *I^2^=*81%, *p*<0.01), 0.72 (95% CI: 0.63–0.79, *I^2^=*63%, *p*<0.01), and 0.99 (95% CI: 0.97–1.00, *I^2^=*0%, *p*=0.71), respectively (**Figures 3A–C**). Four subgroup analyses by study type, number of neoadjuvant therapy cycles, platinum type, and taxane type were performed. No statistically significant differences were observed between subgroup comparisons of CR rate, ORR, and DCR (**Supplementary Figures 4**-**6**).

#### 3.3.3 R0 resection rate

**Figure 4:**
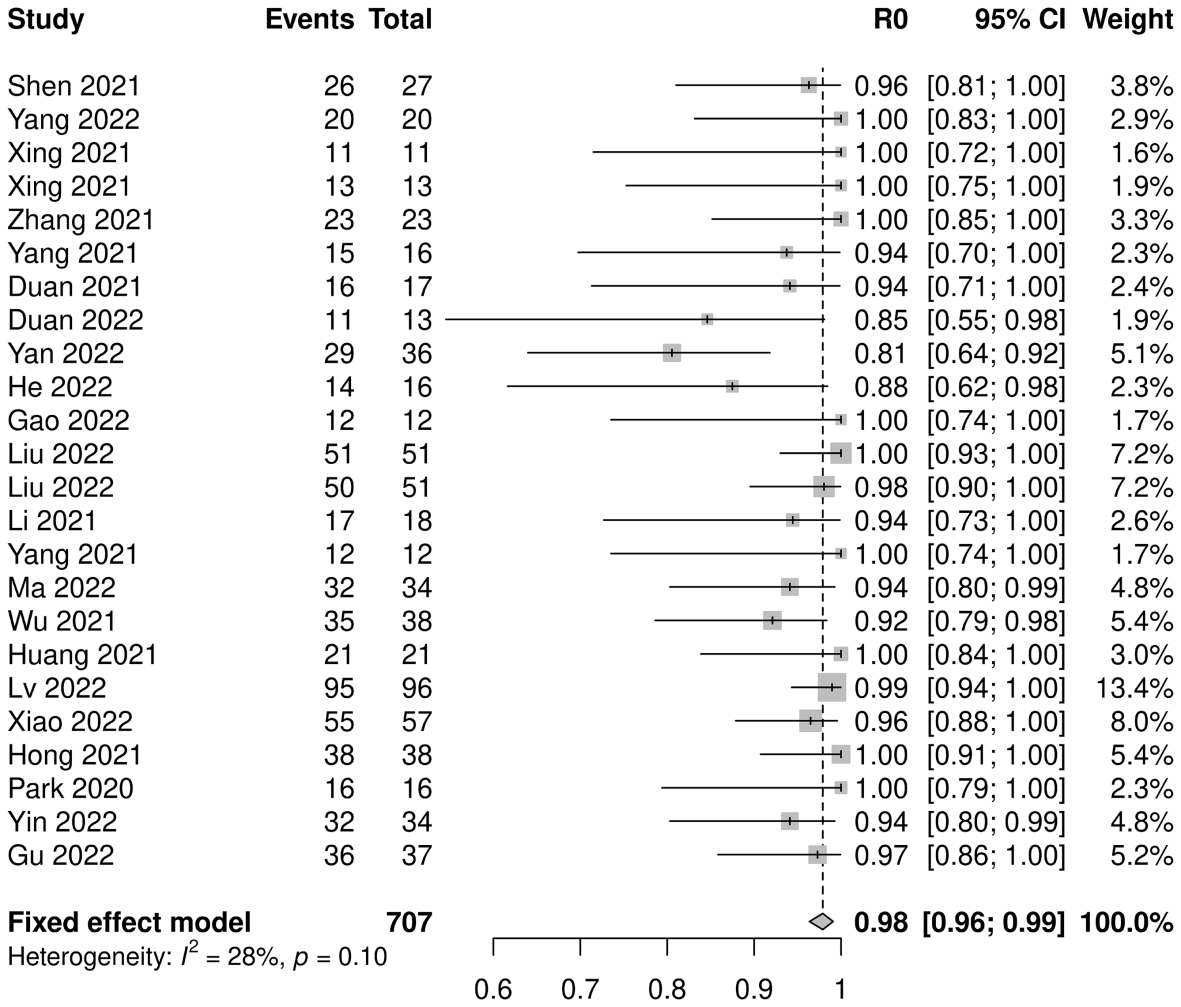
R0 resection rate of neoadjuvant immune checkpoint inhibitors therapy.

Twenty-three studies with 707 patients were pooled for analysis of the R0 resection rate. The combined R0 resection was 0.98 (95% CI: 0.96–0.99, *I^2^=*28%, *p*=0.10) (**Figure 4**). Five subgroup analyses were performed, among which the platinum type showed a statistically significant effect, but not clinically significant, on the R0 resection rate (carboplatin: 0.95, 95% CI: 0.92–0.98; cisplatin: 0.99, 95% CI: 0.97–1.00; *p*=0.01). No significant differences were observed in the other subgroup analyses (**Supplementary Figure 7**).

### 3.4 Safety outcomes

#### 3.4.1 Anastomotic leakage

**Figure 5:**
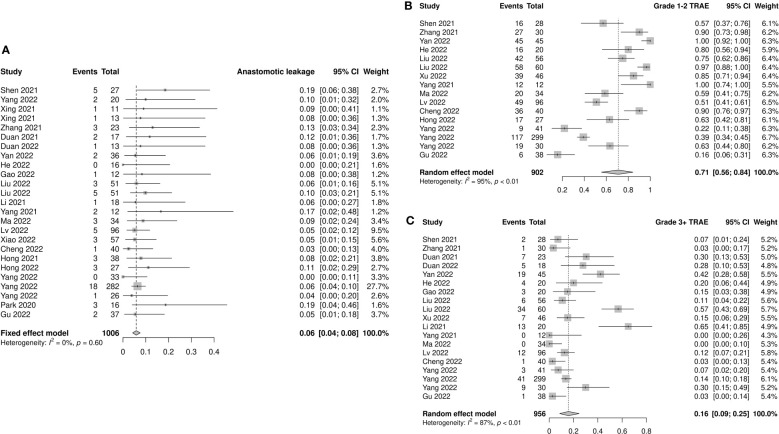
Safety outcomes of neoadjuvant immune checkpoint inhibitors therapy. **(A)** Anastomotic leakage, **(B)** grade 1–2 treatment-related adverse events (TRAEs), and **(C)** grade 3–4 TRAEs.

Anastomotic leakage was reported in 25 studies, which totally included 1006 patients. All studies reported similar low incidences of anastomotic leakage, ranging from 0 to 0.19. The pooled incidence was 0.06 (95% CI: 0.04–0.08, *I^2^=*0%, *p*=0.60; **Figure 5A**). No significant differences were observed in the subgroup analyses (**Supplementary Figure 8**).

#### 3.4.2 Treatment-related adverse events

Fourteen studies provided accessible information on grade 1–2 TRAEs. The incidence of pooled grade 1–2 TRAEs was 0.71 (95% CI: 0.56–0.84, *I^2^=*95%, *p*<0.01; **Figure 5B**). The most frequently reported grade 1–2 TRAEs were leukopenia (8.8%–69.6%), neutropenia (10.4%–65.2%), anemia (8.7%–78.3%), vomiting (6.7%–47.8%), nausea (8.0%–75.0%), and alopecia (8.9%–82.6%). The results of subgroup analysis showed a significant difference between prospective and retrospective studies (prospective: 0.87, 95% CI: 0.74–0.96; retrospective: 0.57, 95% CI: 0.36–0.76; *p*=0.01; **Supplementary Figure 9**).

The incidence of grade 3–4 TRAEs was reported in 17 studies involving 956 patients. The pooled incidence was 0.16 (95% CI: 0.09–0.25, *I^2^=*87%, *p*<0.01; **Figure 5C**). The most frequently reported grade 3–4 TRAEs were leukopenia (3.1%–50.0%), neutropenia (1.8%–39.1%), thrombocytopenia (1.8%–6.7%), and anemia (2.1%–8.7%). Similar to grade 1–2 TRAEs, prospective studies had a higher reported incidence of grade 3–4 TRAEs than retrospective studies (prospective: 0.25, 95% CI: 0.13–0.38; retrospective: 0.07, 95% CI: 0.02–0.14; *p*<0.01). The incidence of grade 3–4 TRAEs in the nICRT group was significantly higher than that in the nICT group (nICRT: 0.46, 95% CI: 0.17–0.77; nICT: 0.14, 95% CI: 0.07–0.22; *p*=0.03). Patients treated with carboplatin had a higher incidence of grade 3–4 TRAEs than those treated with cisplatin (carboplatin: 0.33, 95% CI: 0.15–0.53; cisplatin: 0.04, 95% CI: 0.01–0.09; *p*<0.01). Among the eight studies using carboplatin, grade 3–4 hematologic toxicity TRAEs were reported in six studies, including leukopenia (range: 5%–50%) in five studies, thrombocytopenia (range: 3.6–6.7%) in three studies, and neutropenia (range: 24.4%–39.1%) in two studies. Only three of the six studies using cisplatin mentioned the occurrence of grade 3–4 hematologic toxicity AEs, including leukopenia (range: 1.8%–7.3%), anemia (range: 3.6%–5%), neutropenia (range: 1.8%–5.5%), and thrombocytopenia (1.8%). No significant differences were observed in the subgroup analyses of neoadjuvant treatment cycles and taxane type (**Supplementary Figure 10**).

### 3.5 Sensitivity analysis

For the respective outcomes of pCR, MPR, downstaging rate, CR, ORR, DCR, R0 resection rate, anastomotic leakage, and TRAEs, neither recalculated pooled effect nor statistic inconsistency index (*I^2^*) was largely changed when leaving out each study (**Supplementary Figure 11**), indicating the solid stability of the results from the meta-analysis.

### 3.6 Publication bias

Egger regression tests of pCR, MPR, downstaging rate, CR, ORR, DCR, R0 resection rate, anastomotic leakage, and TRAEs were performed to evaluate publication bias. All the *p* values of Egger’s test were >0.05 (**Supplementary Table 3**), indicating that there are no publication biases in studies synthesized in each analysis. The funnel plots of all items are shown in **Supplementary Figure 12**.

## 4 Discussion

This meta-analysis evaluated the efficacy and safety of neoadjuvant immunotherapy in patients with resectable locally advanced ESCC. The efficacy results showed that the pooled pCR, MPR, and downstaging rates for neoadjuvant immunotherapy were 0.30, 0.56, and 0.74, respectively. The estimated CR, ORR, and DCR rates were 0.14, 0.72, and 0.99, respectively. The pooled R0 resection rate was 0.98. The safety results showed that the incidences of anastomotic leakage, grade 1–2 TRAEs, and grade 3–4 TRAEs were 0.06, 0.71, and 0.16, respectively. These results indicate that immunotherapy achieves good efficacy and safety as a neoadjuvant therapy in patients with ESCC.

In recent years, several studies have confirmed the efficacy and safety of immunotherapy in the second-, first-, and adjuvant treatments of patients with EC. The ATTRACTION-3 (13), KEYNOTE-181 (14), RATIONALE-302 (20), and ESCORT (12) studies have demonstrated the advantage of PD-1 inhibitor monotherapy over chemotherapy as a second-line therapy in patients with EC. The KEYNOTE-590 (15), CheckMate-648 (16), RATIONALE-306 (21), ESCORT-1 (22), JUPITER-06 (23), and ORIENT-15 (24) studies have shown significant benefits of ICI combination with chemotherapy with a manageable safety profile as a first-line treatment in patients with EC. Based on these studies, several PD-1 inhibitors have been approved for first- or second-line treatment of advanced ESCC by the US Food and Drug Administration, European Medicines Agency, and China National Medical Products Administration. In addition, the CheckMate-577 trial showed superior disease-free survival in nivolumab adjuvant treatment of patients with pathological residual disease after nCRT followed by R0 resection. All of these studies have supported the exploration of immunotherapy in the neoadjuvant setting. Recently, several nICRT or nICT trials have consistently demonstrated that immunotherapy can further improve the efficacy of neoadjuvant treatment with acceptable safety profiles in patients with EC. The present meta-analysis showed that the pooled pCR rate of the nICT group was 0.29, which was higher than the previously reported results of nCT (pCR rate: 0.02–0.09) (10, 11, 25–27). The pCR rate of the nICRT group was 0.48, which was slightly higher than that of the nCRT group (pCR rate: 0.28–0.49) (5, 7, 10, 11, 27). Therefore, the findings of this study also suggest the potential advantage of neoadjuvant immunotherapy in patients with ESCC, and neoadjuvant immunotherapy might be a potential standard treatment for patients with ESCC.

The results of the subgroup analyses in this study showed that nICRT achieved a higher pCR rate than nICT; however, the incidence of grade 3–4 TRAEs was higher in the nICRT group than that in the nICT group. In addition, subgroup analysis showed the higher incidence of grade 1–2 and grade 3–4 TRAEs in prospective studies than that in retrospective studies. This might be due to deficient record of AE events and insufficient attention was paid in retrospective studies. These results are similar to those of previous studies that compared nCRT with nCT. However, these trials failed to show a significant survival advantage of nCRT over nCT (10, 11, 27). This situation might be attributed to radiotherapy, leading to serious AEs and delayed death during long-term follow-up. Owing to the lack of long-term follow-up data in the included studies in the current study, we were unable to confirm whether nICRT achieves better long-term survival results than nICT. Considering the efficacy and safety results, nICT may be a more appropriate treatment option for neoadjuvant treatment in patients with ESCC. These results support the widespread clinical use of nICT with ideal efficacy and safety.

Platinum combination therapy with taxanes is a common chemotherapy regimen used in neoadjuvant therapy for ESCC. However, few studies have directly compared the efficacy and safety of carboplatin with those of cisplatin. It is unclear whether nab-paclitaxel has a better efficacy than paclitaxel in ESCC neoadjuvant therapy. In this meta-analysis, subgroup analysis found that the efficacy and safety results were similar when paclitaxel and nab-paclitaxel were used. Moreover, carboplatin did not lead to a significant improvement in efficacy compared with cisplatin. However, the results indicated that carboplatin had a higher rate of grade 3–4 TRAEs than cisplatin did. The results also indicated that carboplatin had higher 3–4 TRAEs than cisplatin. Among 8 studies using carboplatin, grade 3–4 hematologictoxicity TRAEs were reported in 6 studies including leukopenia in 5 studies, thrombocytopenia in 3 studies, neutropenia in 2 studies. While only 3 of the 6 studies using cisplatin mentioned the occurrence of grade 3–4 hematologictoxicity AEs including leukopenia, anemia, neutropenia, and thrombocytopenia. This result was similar to that of previous studies that showed that cisplatin-based chemotherapy led to more frequent nausea, vomiting, and nephrotoxicity in patients with non-small cell lung cancer, whereas grade ≥ 3 thrombocytopenia was more frequently observed in patients receiving carboplatin-based chemotherapy (28–30). In summary, this finding supported the same efficacy of various chemotherapeutic agents when combined with immunotherapy in ESCC neoadjuvant clinical practice. Therefore, individualized chemotherapy regimen for patients is preferable.

The results of the subgroup analyses did not show better efficacy and more toxicity with ≥ 2 cycles of neoadjuvant immunotherapy compared with two cycles. The increased cycle numbers did not bring more benefits, and two cycles may be the ideal number of cycles for neoadjuvant therapy. However, further studies with long-term survival data are required to validate these results.

In addition, the subgroup analysis showed higher incidences of grade 1–2 and 3–4 TRAEs in prospective studies than in retrospective studies. This might be due to the lack of records of AEs and insufficient attention paid in retrospective studies.

However, this study has some limitations. First, most of the included studies were single-arm phase II clinical studies, and phase III RCT studies were lacking. Second, most studies targeted the Asian ESCC population, and the conclusions were only applicable to patients with ESCC. Third, although we used the MINORS to make a quality assessment of the included studies, there was still some acceptable selection bias and heterogeneity among them. Finally, a common problem among the included studies was the lack of long-term survival outcomes, which would take time to wait for the final results.

## 5 Conclusion

Neoadjuvant immunotherapy can achieve good efficacy and safety profiles in patients with ESCC. nICRT may have a higher pCR rate and a higher grade 3–4 TRAEs rate than nICT. Different chemotherapy agents and cycle numbers may have similar efficacy and safety outcomes, except for carboplatin, which has higher incidence of 3–4 TRAEs. In conclusion, this result supports the clinical practice of neoadjuvant immunotherapy in patients with ESCC, and long-term survival data still need to be validated by further studies.

## Author contributions

Conceptualization, WH. Methodology, CW and CL. Resources, XN, HL and JL. Data Curation, NZ, HC, and XM. Date analysis: WH, CW, and CL. Writing-Original Draft Preparation, WH. Writing - Review and Editing, YH, XL, and LP. Supervision, YH, LP and XL. All authors contributed to the article and approved the submitted version.

## Supplementary material

Supplementary Figure 1 Subgroup analyses results of pathological complete response according to **(A)** study type, **(B)** neoadjuvant therapy cycle number, **(C)** combination therapy, **(D)** platinum type, and **(E)** taxanes type.

Supplementary Figure 2 Subgroup analyses results of major pathological response according to **(A)** study type, **(B)** neoadjuvant therapy cycle number, **(C)** platinum type, and **(D)** taxanes type.

Supplementary Figure 3 Subgroup analyses results of downstaging rate according to **(A)** study type, **(B)** neoadjuvant therapy cycle number, **(C)** platinum type, and **(D)** taxanes type.

Supplementary Figure 4 Subgroup analyses results of complete response rate according to **(A)** study type, **(B)** neoadjuvant therapy cycle number, **(C)** platinum type, and **(D)** taxanes type.

Supplementary Figure 5 Subgroup analyses results of objective response rate according to **(A)** study type, **(B)** neoadjuvant therapy cycle number, **(C)** platinum type, and **(D)** taxane type.

Supplementary Figure 6 Subgroup analyses results of disease control rate according to **(A)** study type, **(B)** neoadjuvant therapy cycle number, **(C)** platinum type, and **(D)** taxane type.

Supplementary Figure 7 Subgroup analyses results of R0 resection rate according to **(A)** study type, **(B)** neoadjuvant therapy cycle number, **(C)** combination therapy, **(D)** platinum type, and **(E)** taxane type.

Supplementary Figure 8 Subgroup analyses results of anastomotic leakage incidence according to **(A)** study type, **(B)** neoadjuvant therapy cycle number, **(C)** combination therapy, **(D)** platinum type, and **(E)** taxane type.

Supplementary Figure 9 Subgroup analyses results of grade 1–2 treatment-related adverse event incidence according to **(A)** study type, **(B)** neoadjuvant therapy cycle number, **(C)** platinum type, and **(D)** taxane type.

Supplementary Figure 10 Subgroup analyses results of grade 3–4 treatment-related adverse event incidence according to **(A)** study type, **(B)** neoadjuvant therapy cycle number, **(C)** combination therapy, **(D)** platinum type, and **(E)** taxane type.

Supplementary Figure 11 Sensitivity analysis results of **(A)** pathological complete response, **(B)** major pathological response, **(C)** downstaging rate, **(D)** complete response rate, **(E)** objective response rate, **(F)** disease control rate, **(G)** R0 resection rate, **(H)** incidence of anastomotic leakage, **(I)** grade 1–2 treatment-related adverse events (TRAEs), and **(J)** grade 3–4 TRAEs.

Supplementary Figure 12 Egg’s funnel plot of **(A)** pathological complete response, **(B)** major pathological response, **(C)** downstaging rate, **(D)** complete response rate, **(E)** objective response rate, **(F)** disease control rate, **(G)** R0 resection rate, **(H)** incidence of anastomotic leakage, **(I)** grade 1–2 treatment-related adverse events (TRAEs), and **(J)** grade 3–4 TRAEs.
