# Supplementary material for: The efficacy and safety of neoadjuvant immunotherapy in resectable locally advanced esophageal squamous cell carcinoma: A systematic review and meta-analysis
Source: Front Immunol. 2023 Feb 17;14:1118902. doi: 10.3389/fimmu.2023.1118902 (PMC9981949; doi:10.3389/fimmu.2023.1118902)
Supplement: Supplementary file 1 [file DataSheet_1.docx]

# Supplement Files

**Supplementary Table 1.** Research strategy

| Search strategy for Pubmed: |
| --- |
| ("esophagus"[Title/Abstract] OR "esophageal"[Title/Abstract] OR "oesophageal"[Title/Abstract] OR "gastroesophageal"[Title/Abstract] OR "GEJ"[Title/Abstract] OR "EGJC"[Title/Abstract] OR "ESCC"[Title/Abstract]) AND ("neoadjuvant"[Title/Abstract] OR "preoperative"[Title/Abstract] OR "pre-operative"[Title/Abstract] OR "preoperation"[Title/Abstract] OR "preoperations"[Title/Abstract] OR "pre-operation"[Title/Abstract] OR "pre-operations"[Title/Abstract] OR "presurgical"[Title/Abstract] OR "pre-surgical"[Title/Abstract] OR "presurgery"[Title/Abstract] OR "pre-surgery"[Title/Abstract] OR "preresection"[Title/Abstract] OR "pre-resection"[Title/Abstract] OR "preresectional"[Title/Abstract] OR "before surgery"[Title/Abstract] OR "before resection"[Title/Abstract] OR "before operation"[Title/Abstract] OR "prior to resection"[Title/Abstract] OR "prior to surgery"[Title/Abstract] OR "prior to operation"[Title/Abstract] OR "perioperative"[Title/Abstract] OR "peri operative"[Title/Abstract] OR "perioperation"[Title/Abstract] OR "peri operation"[Title/Abstract] OR "perisurgical"[Title/Abstract] OR "peri surgical"[Title/Abstract] OR "perisurgery"[Title/Abstract] OR "peri surgery"[Title/Abstract] OR "pre-esophagectomy"[Title/Abstract] OR "pre-oesophagectomy"[Title/Abstract]) AND ("immune checkpoint inhibitor"[Title/Abstract] OR "immune checkpoint inhibitors"[Title/Abstract] OR "checkpoint inhibitor"[Title/Abstract] OR "checkpoint inhibitors"[Title/Abstract] OR "checkpoint blockade"[Title/Abstract] OR "checkpoint blockades"[Title/Abstract] OR "programmed cell death protein 1"[Title/Abstract] OR "programmed cell death 1"[Title/Abstract] OR "programmed death 1"[Title/Abstract] OR "programmed death protein 1"[Title/Abstract] OR "PD-1"[Title/Abstract] OR "PD1"[Title/Abstract] OR "programmed cell death receptor-1 inhibitor"[Title/Abstract] OR "programmed cell death receptor-1 inhibitors"[Title/Abstract] OR "programmed cell death 1 receptor inhibitor"[Title/Abstract] OR "programmed cell death 1 receptor inhibitors"[Title/Abstract] OR "anti-pd-1 antibody"[Title/Abstract] OR "anti-pd-1 antibodies"[Title/Abstract] OR "tislelizumab"[Title/Abstract] OR "BGB-A317"[Title/Abstract] OR "pembrolizumab"[Title/Abstract] OR "Keytruda"[Title/Abstract] OR "MK-3475"[Title/Abstract] OR "nivolumab"[Title/Abstract] OR "Opdivo"[Title/Abstract] OR "BMS-936558"[Title/Abstract] OR "toripalimab"[Title/Abstract] OR "JS-001"[Title/Abstract] OR "sintilimab"[Title/Abstract] OR "IBI308"[Title/Abstract] OR "camrelizumab"[Title/Abstract] OR "SHR-1210"[Title/Abstract] OR "cemiplimab"[Title/Abstract] OR "REGN2810"[Title/Abstract] OR "penpulimab"[Title/Abstract] OR "AK105"[Title/Abstract] OR "dostarlimab"[Title/Abstract] OR "TSR-042"[Title/Abstract] OR "zimberelimab"[Title/Abstract] OR "GLS-010"[Title/Abstract] OR "spartalizumab"[Title/Abstract] OR "PDR001"[Title/Abstract] OR "prolgolimab"[Title/Abstract] OR "BCD-100"[Title/Abstract] OR "retifanlimab"[Title/Abstract] OR "sasanlimab"[Title/Abstract] OR "PF-06801591"[Title/Abstract] OR "balstilimab"[Title/Abstract] OR "AGEN2034"[Title/Abstract] OR "budigalimab"[Title/Abstract] OR "ABBV-181"[Title/Abstract] OR "pucotenlimab"[Title/Abstract] OR "AK103"[Title/Abstract] OR "serplulimab"[Title/Abstract] OR "HLX10"[Title/Abstract] OR "programmed cell death 1 ligand 1"[Title/Abstract] OR "programmed death 1 ligand 1"[Title/Abstract] OR "programmed death ligand 1"[Title/Abstract] OR "PD-L1"[Title/Abstract] OR "PDL1"[Title/Abstract] OR "anti-pd-l1 antibody"[Title/Abstract] OR "anti-pd-l1 antibodies"[Title/Abstract] OR "anti-pd-l1 inhibitor"[Title/Abstract] OR "anti-pd-l1 inhibitors"[Title/Abstract] OR "atezolizumab"[Title/Abstract] OR "Tecentriq"[Title/Abstract] OR "MPDL3280A"[Title/Abstract] OR "avelumab"[Title/Abstract] OR "Bavencio"[Title/Abstract] OR "durvalumab"[Title/Abstract] OR "Imfinzi"[Title/Abstract] OR "MEDI4736"[Title/Abstract] OR "adebrelimab"[Title/Abstract] OR "SHR-1316"[Title/Abstract] OR "envafolimab"[Title/Abstract] OR "KN035"[Title/Abstract] OR "sugemalimab"[Title/Abstract] OR "CS1001"[Title/Abstract] OR "Immunotherapy"[Title/Abstract] OR "Immuno-therapy"[Title/Abstract] OR "Immune therapy"[Title/Abstract] OR "immunotherapic"[Title/Abstract] OR "immunogenic"[Title/Abstract] OR "immunological"[Title/Abstract] OR "immuno-oncology"[Title/Abstract]) |
| Search strategy for Embase |
| (('immune checkpoint inhibitor':ab,ti OR 'immune checkpoint inhibitors':ab,ti OR 'checkpoint inhibitor':ab,ti OR 'checkpoint inhibitors':ab,ti OR 'checkpoint blockade':ab,ti OR 'checkpoint blockades':ab,ti OR 'programmed cell death protein 1':ab,ti OR 'programmed cell death 1':ab,ti OR 'programmed death 1':ab,ti OR 'programmed death protein 1':ab,ti OR 'pd-1':ab,ti OR pd1:ab,ti OR 'programmed cell death receptor-1 inhibitor':ab,ti OR 'programmed cell death receptor-1 inhibitors':ab,ti OR 'programmed cell death 1 receptor inhibitor':ab,ti OR 'programmed cell death 1 receptor inhibitors':ab,ti OR 'anti-pd-1 antibody':ab,ti OR 'anti-pd-1 antibodies':ab,ti OR tislelizumab:ab,ti OR 'bgb-a317':ab,ti OR pembrolizumab:ab,ti OR keytruda:ab,ti OR 'mk-3475':ab,ti OR nivolumab:ab,ti OR opdivo:ab,ti OR 'bms-936558':ab,ti OR toripalimab:ab,ti OR 'js-001':ab,ti OR sintilimab:ab,ti OR ibi308:ab,ti OR camrelizumab:ab,ti OR 'shr-1210':ab,ti OR cemiplimab:ab,ti OR regn2810:ab,ti OR penpulimab:ab,ti OR ak105:ab,ti OR dostarlimab:ab,ti OR 'tsr-042':ab,ti OR zimberelimab:ab,ti OR 'gls-010':ab,ti OR spartalizumab:ab,ti OR pdr001:ab,ti OR prolgolimab:ab,ti OR 'bcd 100':ab,ti OR retifanlimab:ab,ti OR mga012:ab,ti OR sasanlimab:ab,ti OR 'pf-06801591':ab,ti OR balstilimab:ab,ti OR agen2034:ab,ti OR budigalimab:ab,ti OR 'abbv-181':ab,ti OR genolimzumab:ab,ti OR 'gb-226':ab,ti OR pucotenlimab:ab,ti OR ak103:ab,ti OR serplulimab:ab,ti OR hlx10:ab,ti OR 'programmed cell death 1 ligand 1':ab,ti OR 'programmed death 1 ligand 1':ab,ti OR 'programmed death ligand 1':ab,ti OR 'pd-l1':ab,ti OR pdl1:ab,ti OR 'anti-pd-l1 antibody':ab,ti OR 'anti-pd-l1 antibodies':ab,ti OR 'anti-pd-l1 inhibitor':ab,ti OR 'anti-pd-l1 inhibitors':ab,ti OR atezolizumab:ab,ti OR tecentriq:ab,ti OR mpdl3280a:ab,ti OR avelumab:ab,ti OR bavencio:ab,ti OR durvalumab:ab,ti OR imfinzi:ab,ti OR medi4736:ab,ti OR adebrelimab:ab,ti OR 'shr-1316':ab,ti OR envafolimab:ab,ti OR kn035:ab,ti OR 'kl-a167':ab,ti OR dewallumab:ab,ti OR 'sti-a1014':ab,ti OR sugemalimab:ab,ti OR cs1001:ab,ti OR 'immunotherapy':ab,ti OR 'immuno-therapy':ab,ti OR 'immune therapy':ab,ti OR immunotherapic:ab,ti OR immunogenic:ab,ti OR immunological:ab,ti OR 'immuno-oncology':ab,ti) AND ('neoadjuvant therapy'/mj OR 'neoadjuvant treatment'/mj OR 'perioperative period'/mj OR neoadjuvant:ab,ti OR preoperative:ab,ti OR 'pre-operative':ab,ti OR preoperation:ab,ti OR preoperations:ab,ti OR 'pre-operation':ab,ti OR 'pre-operations':ab,ti OR presurgical:ab,ti OR 'pre-surgical':ab,ti OR presurgery:ab,ti OR 'pre-surgery':ab,ti OR preresection:ab,ti OR 'pre-resection':ab,ti OR preresectional:ab,ti OR 'before surgery':ab,ti OR 'before resection':ab,ti OR 'before operation':ab,ti OR perioperative:ab,ti OR 'peri operative':ab,ti OR perioperation:ab,ti OR 'peri operation':ab,ti OR perisurgical:ab,ti OR 'peri surgical':ab,ti OR perisurgery:ab,ti OR 'peri surgery':ab,ti OR 'peri section':ab,ti OR perisection:ab,ti OR perioperations:ab,ti OR perisurgeries:ab,ti OR 'peri surgeries':ab,ti) AND (esophagus:ab,ti OR esophageal:ab,ti OR oesophageal:ab,ti OR gastroesophageal:ab,ti OR gej:ab,ti OR egjc:ab,ti OR escc:ab,ti)) AND ('article'/it OR 'article in press'/it OR 'chapter'/it OR 'editorial'/it OR 'erratum'/it OR 'note'/it OR 'preprint'/it OR 'review'/it OR 'short survey'/it OR 'tombstone'/it) |
| Search strategy for Cochrane Library |
| (esophagus OR esophageal OR oesophageal OR gastroesophageal OR GEJ OR EGJC OR ESCC):ti,ab,kw AND (neoadjuvant OR preoperative OR 'pre-operative' OR preoperation OR preoperations OR 'pre-operation' OR 'pre-operations' OR presurgical OR 'pre-surgical' OR presurgery OR 'pre-surgery' OR preresection OR 'pre-resection' OR preresectional OR 'before surgery' OR 'before resection' OR 'before operation' OR perioperative OR 'peri operative' OR perioperation OR 'peri operation' OR perisurgical OR 'peri surgical' OR perisurgery OR 'peri surgery' OR 'peri section' OR perisection OR perioperations OR perisurgeries OR 'peri surgeries'):ti,ab,kw AND ('immune checkpoint inhibitor' OR 'immune checkpoint inhibitors' OR 'checkpoint inhibitor' OR 'checkpoint inhibitors' OR 'checkpoint blockade' OR 'checkpoint blockades' OR 'programmed cell death protein 1' OR 'programmed cell death 1' OR 'programmed death 1' OR 'programmed death protein 1' OR 'PD-1' OR PD1 OR 'programmed cell death receptor-1 inhibitor' OR 'programmed cell death receptor-1 inhibitors' OR 'programmed cell death 1 receptor inhibitor' OR 'programmed cell death 1 receptor inhibitors' OR 'anti-pd-1 antibody' OR 'anti-pd-1 antibodies' OR tislelizumab OR 'BGB-A317' OR pembrolizumab OR Keytruda OR 'MK-3475' OR nivolumab OR Opdivo OR 'BMS-936558' OR toripalimab OR 'JS-001' OR sintilimab OR IBI308 OR camrelizumab OR 'SHR-1210' OR cemiplimab OR REGN2810 OR penpulimab OR AK105 OR dostarlimab OR 'TSR-042' OR zimberelimab OR 'GLS-010' OR spartalizumab OR PDR001 OR prolgolimab OR BCD-100 OR retifanlimab OR MGA012 OR sasanlimab OR 'PF-06801591' OR balstilimab OR AGEN2034 OR budigalimab OR 'ABBV-181' OR genolimzumab OR 'GB-226' OR pucotenlimab OR AK103 OR serplulimab OR HLX10 OR 'programmed cell death 1 ligand 1' OR 'programmed death 1 ligand 1' OR 'programmed death ligand 1' OR 'PD-L1' OR PDL1 OR 'anti-pd-l1 antibody' OR 'anti-pd-l1 antibodies' OR 'anti-pd-l1 inhibitor' OR 'anti-pd-l1 inhibitors' OR atezolizumab OR Tecentriq OR MPDL3280A OR avelumab OR Bavencio OR durvalumab OR Imfinzi OR MEDI4736 OR adebrelimab OR 'SHR-1316' OR envafolimab OR KN035 OR 'kl-a167' OR dewallumab OR 'STI-A1014' OR sugemalimab OR CS1001 OR 'Immunotherapy' OR 'Immuno-therapy' OR 'Immune therapy' OR immunotherapic OR immunogenic OR immunological OR 'immuno-oncology'):ti,ab,kw |

**Supplementary Table 2.** Quality assessment of include studies.

| **Studies ID** | **Study types** | **A clearly stated aim** | **Inclusion of consecutive patients** | **Prospective collection of data** | **Endpoints appropriated to the aim of the study** | **Unbiased assessment of the study endpoint** | **Follow-up period appropriate to the aim of the study** | **Loss to follow up less than 5%** | **Prospective calculation of the study size** | **An adequate control group** | **Contemporary groups** | **Baseline equivalence of groups** | **Adequate statistical analyses** | **Total** |
| --- | --- | --- | --- | --- | --- | --- | --- | --- | --- | --- | --- | --- | --- | --- |
| Shen 2021 | Non-comparative prospective | 2 | 2 | 2 | 2 | 2 | 2 | 2 | 0 | - | - | - | - | 14 |
| Yang 2022 | Non-comparative prospective | 2 | 2 | 2 | 2 | 2 | 2 | 2 | 0 | - | - | - | - | 14 |
| Xing 2021 | Comparative prospective | 2 | 2 | 2 | 2 | 2 | 0 | 0 | 2 | 1 | 2 | 2 | 2 | 19 |
| Zhang 2021 | Non-comparative prospective | 2 | 2 | 2 | 2 | 2 | 1 | 1 | 1 | - | - | - | - | 13 |
| Yang 2021 | Non-comparative prospective | 2 | 2 | 2 | 2 | 2 | 2 | 2 | 0 | - | - | - | - | 14 |
| Duan 2021 | Non-comparative prospective | 2 | 2 | 2 | 2 | 2 | 2 | 1 | 2 | - | - | - | - | 15 |
| Duan 2022 | Non-comparative prospective | 2 | 2 | 2 | 2 | 2 | 2 | 2 | 0 | - | - | - | - | 14 |
| Yan 2022 | Non-comparative prospective | 2 | 2 | 2 | 2 | 2 | 0 | 0 | 2 | - | - | - | - | 12 |
| He 2022 | Non-comparative prospective | 2 | 2 | 2 | 2 | 2 | 0 | 0 | 0 | - | - | - | - | 10 |
| Gao 2022 | Non-comparative prospective | 2 | 2 | 2 | 2 | 2 | 0 | 0 | 0 | - | - | - | - | 10 |
| Liu 2022 | Non-comparative prospective | 2 | 2 | 2 | 2 | 2 | 0 | 0 | 2 | - | - | - | - | 12 |
| Liu 2022 | Non-comparative prospective | 2 | 2 | 2 | 2 | 2 | 0 | 0 | 2 | - | - | - | - | 12 |
| Xu 2022 | Non-comparative prospective | 2 | 2 | 2 | 2 | 2 | 0 | 0 | 2 | - | - | - | - | 12 |
| Li 2021 | Non-comparative prospective | 2 | 2 | 2 | 2 | 2 | 1 | 2 | 1 | - | - | - | - | 14 |
| Yang 2021 | Non-comparative retrospective | 2 | 2 | 2 | 2 | 2 | 0 | 0 | 0 | - | - | - | - | 10 |
| Ma 2022 | Non-comparative retrospective | 2 | 2 | 2 | 2 | 2 | 2 | 1 | 0 | - | - | - | - | 13 |
| Wu 2021 | Non-comparative retrospective | 2 | 1 | 2 | 2 | 2 | 0 | 0 | 0 | - | - | - | - | 9 |
| Zhang 2022 | Non-comparative retrospective | 2 | 2 | 2 | 2 | 2 | 0 | 0 | 0 | - | - | - | - | 10 |
| Huang 2021 | Comparative retrospective | 2 | 2 | 2 | 2 | 2 | 0 | 0 | 0 | 2 | 2 | 2 | 2 | 18 |
| Lv 2022 | Non-comparative retrospective | 2 | 2 | 2 | 2 | 2 | 2 | 1 | 0 | - | - | - | - | 13 |
| Xiao 2022 | Comparative retrospective | 2 | 2 | 2 | 2 | 2 | 2 | 2 | 0 | 2 | 2 | 2 | 2 | 22 |
| Yu 2022 | Non-comparative retrospective | 2 | 2 | 2 | 2 | 2 | 0 | 0 | 0 | - | - | - | - | 10 |
| Cheng 2022 | Comparative retrospective | 2 | 2 | 2 | 2 | 2 | 0 | 0 | 0 | 2 | 2 | 2 | 2 | 18 |
| Hong 2021 | Comparative retrospective | 2 | 2 | 2 | 2 | 2 | 0 | 0 | 0 | 2 | 2 | 2 | 2 | 18 |
| Hong 2022 | Comparative retrospective | 2 | 2 | 2 | 2 | 2 | 0 | 0 | 0 | 2 | 2 | 2 | 2 | 18 |
| Zhou 2022 | Non-comparative retrospective | 2 | 2 | 2 | 2 | 2 | 1 | 2 | 0 | - | - | - | - | 13 |
| Yang 2022 | Comparative retrospective | 2 | 2 | 2 | 2 | 2 | 0 | 0 | 0 | 0 | 2 | 2 | 2 | 16 |
| Park 2020 | Comparative retrospective | 2 | 2 | 2 | 2 | 2 | 1 | 0 | 0 | 2 | 2 | 2 | 2 | 19 |
| Yin 2022 | Non-comparative retrospective | 2 | 2 | 2 | 2 | 2 | 2 | 2 | 0 | - | - | - | - | 14 |
| Gu 2022 | Non-comparative retrospective | 2 | 2 | 2 | 2 | 2 | 0 | 0 | 0 | - | - | - | - | 10 |

**Supplementary Table 3.** *P* value of Egger’s test

| **Item** | ***P* value** |
| --- | --- |
| pCR | 0.543 |
| MPR | 0.431 |
| R0 | 0.281 |
| CR | 0.370 |
| ORR | 0.326 |
| DCR | 0.160 |
| Downstaging | 0.632 |
| Anastomotic leakage | 0.052 |
| Grade 1-2 TRAEs | 0.053 |
| Grade 3-4 TRAEs | 0.729 |
