# Supplementary figures and images for: The efficacy and safety of neoadjuvant immunotherapy in resectable locally advanced esophageal squamous cell carcinoma: A systematic review and meta-analysis
Source: Front Immunol. 2023 Feb 17;14:1118902. doi: 10.3389/fimmu.2023.1118902 (PMC9981949; doi:10.3389/fimmu.2023.1118902)

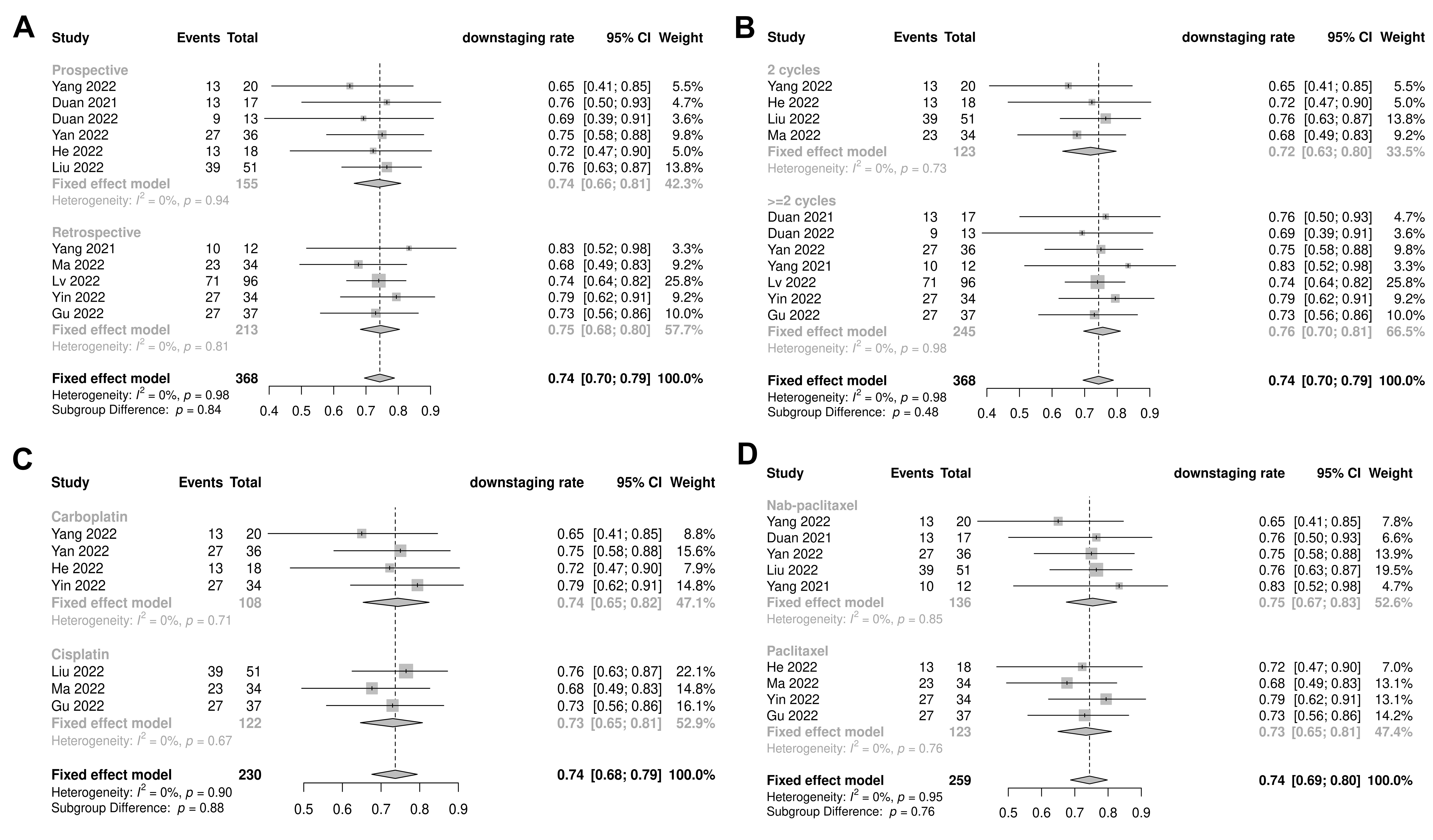

Supplement: Supplementary Figure 3 — Subgroup analyses results of downstaging rate according to (A) study type, (B) neoadjuvant therapy cycle number, (C) platinum type, and (D) taxanes type. [file Image_3.tif]

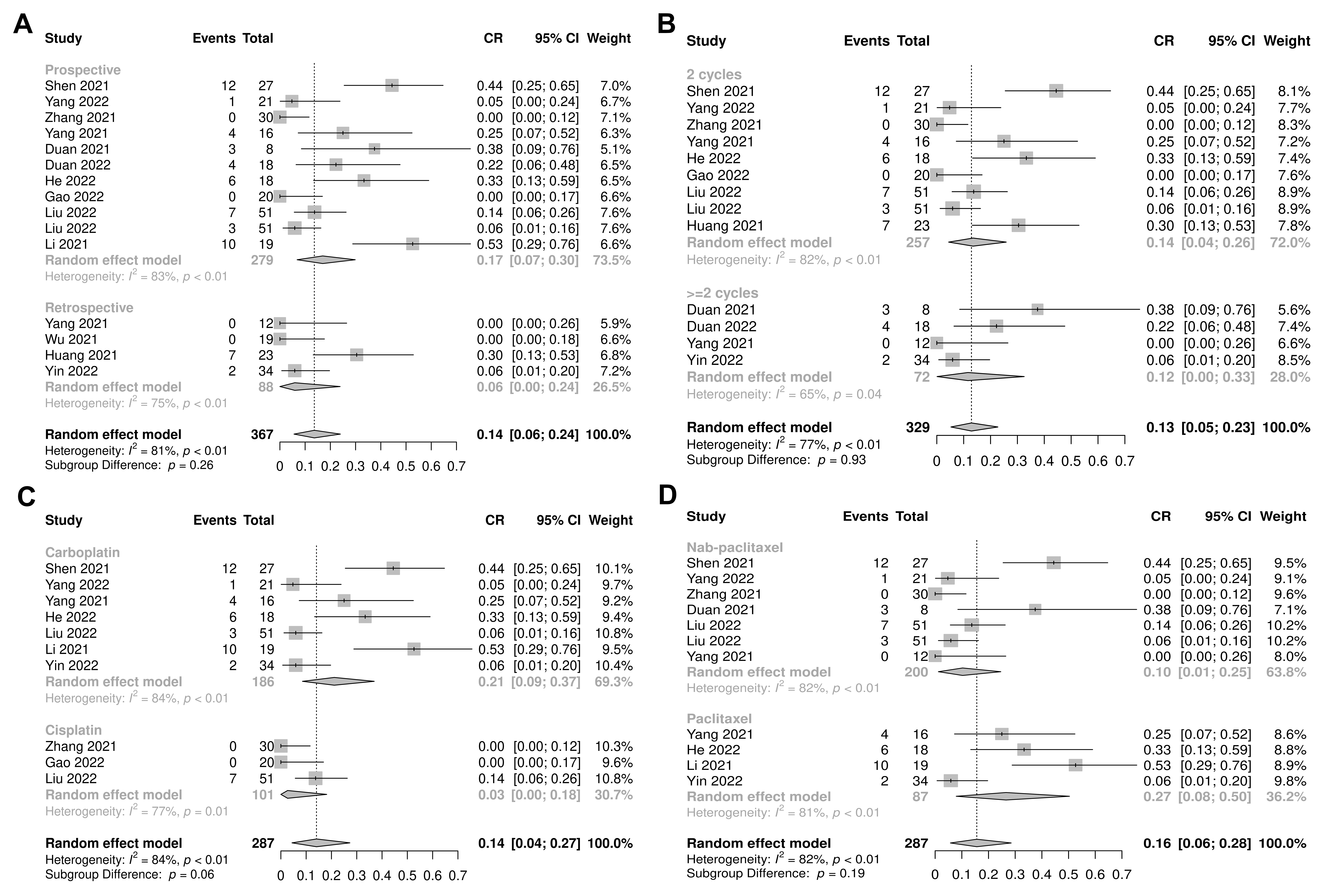

Supplement: Supplementary Figure 4 — Subgroup analyses results of complete response rate according to (A) study type, (B) neoadjuvant therapy cycle number, (C) platinum type, and (D) taxanes type. [file Image_4.tif]
